# A very large perpendicular magnetic anisotropy in Pt/Co/MgO trilayers fabricated by controlling the MgO sputtering power and its thickness

**DOI:** 10.1038/s41598-018-19656-9

**Published:** 2018-01-19

**Authors:** Hyung Keun Gweon, Seok Jin Yun, Sang Ho Lim

**Affiliations:** Department of Materials Science and Engineering, Korea University, Seoul, 02841 Korea

## Abstract

The perpendicular magnetic anisotropy (PMA) properties of Pt/Co/MgO trilayers are investigated as a function of the MgO sputtering power (*P*_MgO_) and its thickness (*t*_MgO_), both of which are important parameters affecting the degree of oxygen interpenetration into Co during sputtering. A strong PMA is achieved at small values of *P*_MgO_ and *t*_MgO_, where the oxygen interpenetration into Co is expected to be small. The range of oxygen interpenetration is relatively extended in such a way that it affects both the Pt/Co and Co/MgO interfaces. The PMA properties of as-deposited samples are improved by post-annealing for temperatures up to 400 °C examined in this study, probably due to the diffusion of the interpenetrated oxygen atoms toward the Co/MgO interface. In a structure of Pt/Co (0.6 nm)/MgO (2 nm), which is fabricated at *P*_MgO_ = 50 W and then annealed at 400 °C, a huge saturation field is achieved (over 40 kOe) indicating a very strong PMA. Between the two interfaces of Pt/Co and Co/MgO, the PMA is mainly due to the former in the as-deposited state, but the contribution of the latter increases with the increase in the annealing temperature.

## Introduction

The magnetic thin-film structure of heavy-metal/ferromagnet/oxide has been intensively investigated owing to its practical importance in the application of magnetic random access memory (MRAM) devices and the theoretical interest in understanding spin-orbit coupling (SOC) effects. Particular focus has been on the Pt/Co/MO_*x*_ structures (M = Al or Mg), which were found to exhibit strong spin-orbit torque effects^1–4^, Dzyaloshinskii-Moriya interactions^5–7^, and perpendicular magnetic anisotropy (PMA)^8–12^. Among these properties, a strong PMA is essential toward the realization of high-density MRAM, and therefore, studies on this subject have been extensively carried out^13^. Previous studies showed that the PMA in these stacks stems from the interfacial effects, with its characteristics being very sensitive to the quality of the interfaces^14^. In Pt/Co/MO_*x*_ structures, there are two types of interfaces responsible for the PMA: bottom Pt/Co and top Co/MO_*x*_. The PMA from the former is due to the hybridization between Co and Pt having a strong SOC. However, the PMA from the latter is attributed to the charge transfer between Co and oxygen, which causes an increase in the band splitting of the hybridized band levels (*d*_*z*_2, *d*_*xz*_, *d*_*yz*_, *p*_*z*_) along the out-of-plane magnetization direction^15^. There were many reports in the literature showing the role of oxygen in forming the PMA at this top interface. In the cases where a full metallic stack of Pt/Co/Al was deposited and subsequently plasma-oxidized, the strength of PMA was affected very sensitively by the conditions of plasma oxidation^9,12^. This means that the PMA is sensitive to the degree of oxidation near the Co/AlO_*x*_ interface. Although a strong PMA can be achieved under an optimal condition of plasma oxidation, the process is rather complex and has a problem of excessive target contamination, and furthermore, the window showing the optimum properties is narrow^9,16^. A better way to overcome these problems is to deposit an oxide layer directly, rather than to deposit a metal precursor followed by plasma oxidation^17–19^. In this case, sputtering conditions during the deposition of an oxide layer can be used to control the degree of oxidation near the Co/MO_*x*_ interface. The stack structure examined in this study is Pt/Co/MgO, with the choice of MgO being obvious due to its high tunnelling magnetoresistance^20^. The parameters used were the sputtering power during the deposition of the MgO layer (*P*_MgO_) and its thickness (*t*_MgO_). An MgO-free model stack of Pt/Co/Cu was also used for a comparative study, where the element Cu was chosen to replace MgO due to the immiscibility of Co and Cu^21–23^.

## Results

### Effects of *P*_MgO_ and *t*_MgO_ on PMA properties

**Figure 1:**
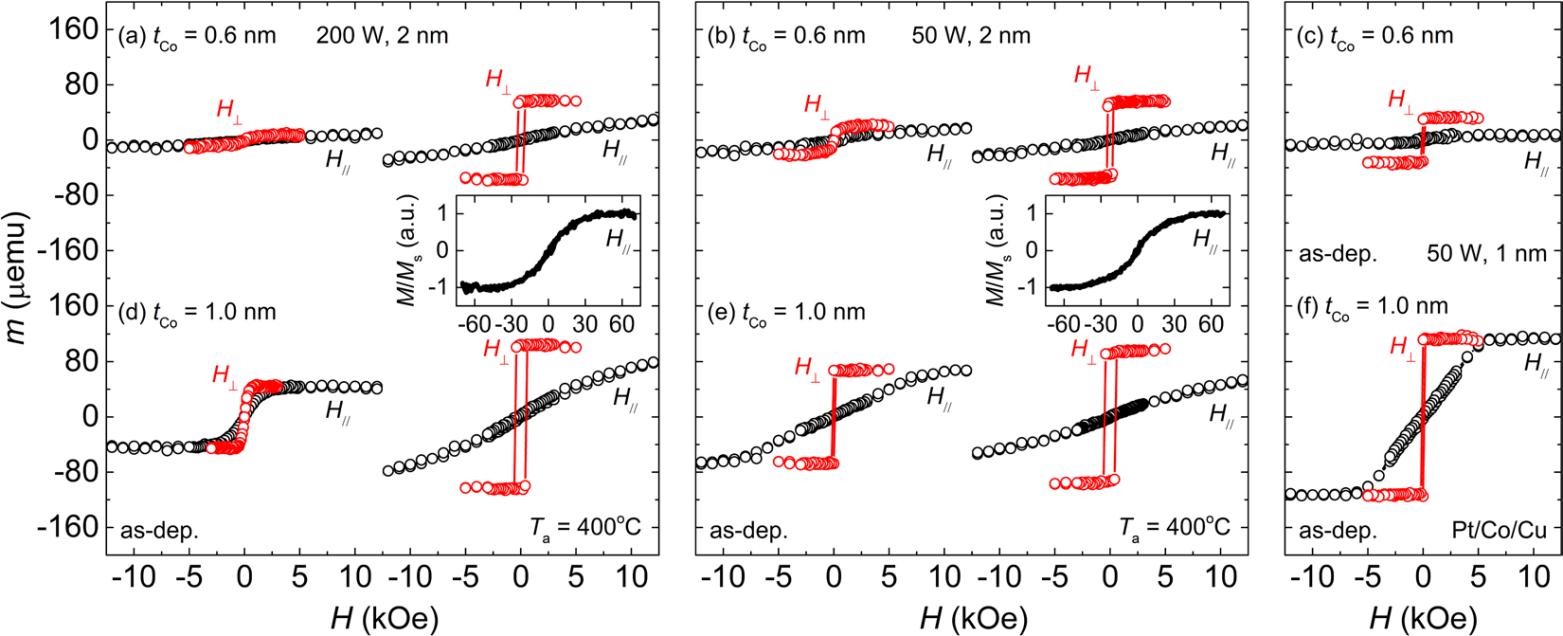
Out-of-plane (*H*_⊥_) and in-plane (*H*_//_) *m*-*H* loops for *t*_Co_ = 0.6 nm (upper panels (a–c)) and 1.0 nm (lower panels (d–f)). The left panel ((**a**) and (**d**)) and middle panel ((**b**) and (**e**)) represent *m*-*H* loops for the Pt/Co/MgO samples at (200, 2) and (50, 2), respectively. Within the panel, two sets of results are shown—one in the as-deposited state and the other after annealing at 400 °C. The insets in (**a**) and (**b**) are the in-plane *m*-*H* loops obtained from the SQUID measurements. The right panel is for comparison, where (**c**) and (**f**) are the *m*-*H* loops for the samples of Pt/Co/MgO at (50, 1) and of Pt/Co/Cu, respectively, in the as-deposited state.

The main process variables in this study are *P*_MgO_ (50 and 200 W) and *t*_MgO_ (1 and 2 nm), because they are considered to greatly affect the oxygen penetration into Co during the deposition of MgO, and subsequently, the PMA properties^24^. Therefore, these two parameters are conveniently denoted as (*P*_MgO_, *t*_MgO_). Figure 1(a–f) show *m*-*H* hysteresis loops (where *m* and *H* denote the magnetic moment and applied magnetic field, respectively) of some typical samples measured along the out-of-plane (*H*_⊥_) and in-plane (*H*_//_) directions. The results are for the Co layer thickness (*t*_Co_) of 0.6 nm (upper panels, (a–c)) and 1.0 nm (lower panels, (d–f)). The left ((a) and (d)) and middle ((b) and (e)) panels show the results for the samples fabricated at the conditions of (200, 2) and (50, 2), respectively. For each sample, two sets of results are shown—one for the samples in the as-deposited state and the other after annealing at 400 °C. The right panel shows the results for the sample fabricated at (50, 1) ((c)) and also for the MgO-free model stack of Pt/Co (1 nm)/Cu ((f)), both samples being in the as-deposited state. For the as-deposited Pt/Co (0.6 nm)/MgO samples (upper panels), the PMA is not clearly visible for the sample at (200, 2) but it is clearly formed for the samples at (50, 2) and (50, 1), with the latter showing the strongest PMA. For the as-deposited sample at (200, 2) in particular, the magnetization is very small, and furthermore, the magnetization behaviour appears to be superparamagnetic^9^, indicated by the fact that no substantial difference occurs between the in-plane and out-of-plane loops and both the remanence and the coercivity are close to zero. These results strongly indicate a greater interpenetration of oxygen into Co during the deposition of MgO at higher values of *P*_MgO_ and *t*_MgO_.

**Figure 2:**
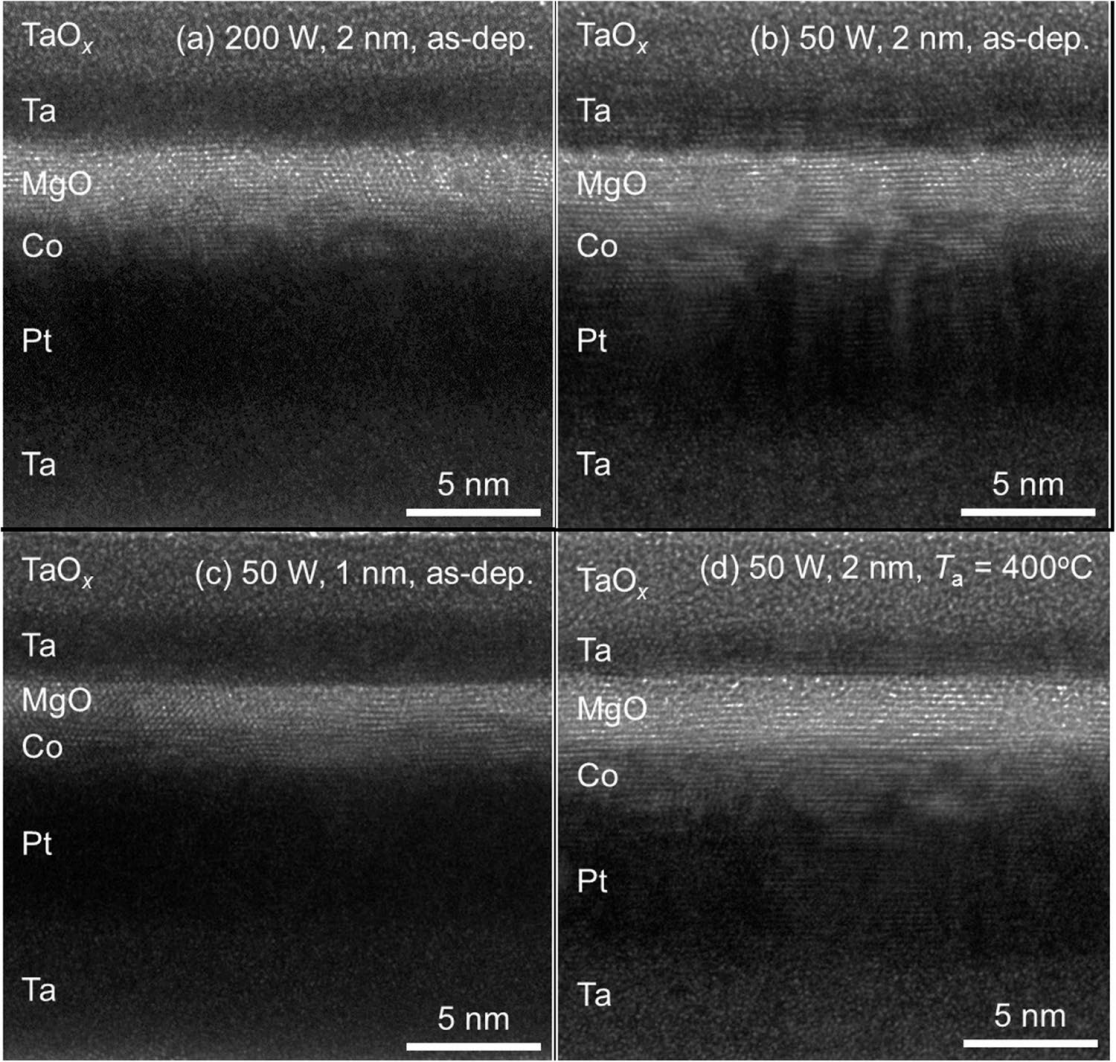
Cross-sectional HRTEM images for the as-deposited Pt/Co (2 nm)/MgO samples fabricated at (**a**) (200, 2), (**b**) (50, 2), and (**c**) (50, 1), and (**d**) for the sample with the same structure fabricated at (50, 2) and then annealed at 400 °C.

In the present Pt/Co/MgO stack, the PMA results from the two interfaces of Pt/Co and Co/MgO. Between these two, the former should be less affected by the oxygen interpenetration than the latter, where it is expected that the oxygen contamination is substantial, and furthermore, its geometry in the as-deposited state is quite blurred^19^, thus making its PMA contribution negligible. This is confirmed by cross-sectional high-resolution transmission electron microscopy (HRTEM), its images being shown in Fig. 2(a–d) for the as-deposited Pt/Co (2 nm)/MgO samples fabricated at (200, 2), (50, 2), and (50, 1), and for the sample with the same structure fabricated at (50, 2) and then annealed at 400 °C, respectively. It is seen from the images that the Co/MgO interface in the as-deposited state is quite blurred, which is particularly true for the samples fabricated at (200, 2) and (50, 2), indicative of the interpenetration of oxygen atoms during the deposition of MgO. Although the Co/MgO interface of as-deposited samples is quite blurred in general, the Co/MgO interface of the as-deposited sample at (50, 1) appears clearer than that of the samples at (200, 2) and (50, 2). This implies that the PMA in the as-deposited state mainly results from the Pt/Co interface and the change in its properties depending on the fabrication condition is due to the change in the degree of oxygen contamination at this Pt/Co interface. It is noted that the quality of the Co/MgO interface improves upon annealing, which is seen clearly from a comparison of the results in Fig. 2(b) and (d) for the same sample fabricated at (50, 2).

The level of oxygen contamination at the Pt/Co interface is expected to be higher when the values of *P*_MgO_ and *t*_MgO_ are higher and the *t*_Co_ value is lower. This expectation is validated by the observed results for the as-deposited samples at the conditions of (200, 2), (50, 2), and (50, 1). (Fig. 1(a–e)) The effects of *t*_Co_ on the oxygen contamination of the Pt/Co interface and the resultant PMA properties can be seen clearly by comparing the results for the samples with *t*_Co_ = 0.6 nm (upper panels) and *t*_Co_ = 1.0 nm (lower panels). For example, at the same condition of (200, 2), the PMA is not visible at *t*_Co_ = 0.6 nm (Fig. 1(a)), but it is clearly formed at *t*_Co_ = 1.0 nm (Fig. 1(d)), though it is still weak. A similar behaviour is observed for the samples fabricated at (50, 2); the PMA is clearly formed at *t*_Co_ = 0.6 and 1.0 nm, but the PMA strength of the latter is significantly greater than that of the former. (Fig. 1(b) and (e)) In addition to *t*_Co_, *P*_MgO_ also plays an important role in affecting the PMA properties. This is seen clearly by comparing the PMA properties of the as-deposited Pt/Co (1 nm)/MgO samples fabricated at different *P*_MgO_ values with those of the Pt/Co (1 nm)/Cu. The anisotropy fields (*H*_Keff_) are 2.4, 5.6, and 9.9 kOe for the stacks of Pt/Co/MgO at (200, 2), Pt/Co/Cu, and Pt/Co/MgO at (50, 2), respectively. This indicates that the PMA of the Pt/Co/Cu is significantly stronger than that of the Pt/Co/MgO at (200, 2), but it is weaker than that of the Pt/Co/MgO at (50, 2). Considering that the PMA of as-deposited Pt/Co/MgO samples is due to the bottom Pt/Co interface, the Pt/Co interface of the Pt/Co/MgO sample at (200, 2) is contaminated by interpenetrated oxygen atoms to a greater extent than that of Pt/Co/MgO at (50, 2).

### Some evidence of oxygen interpenetration

**Figure 3:**
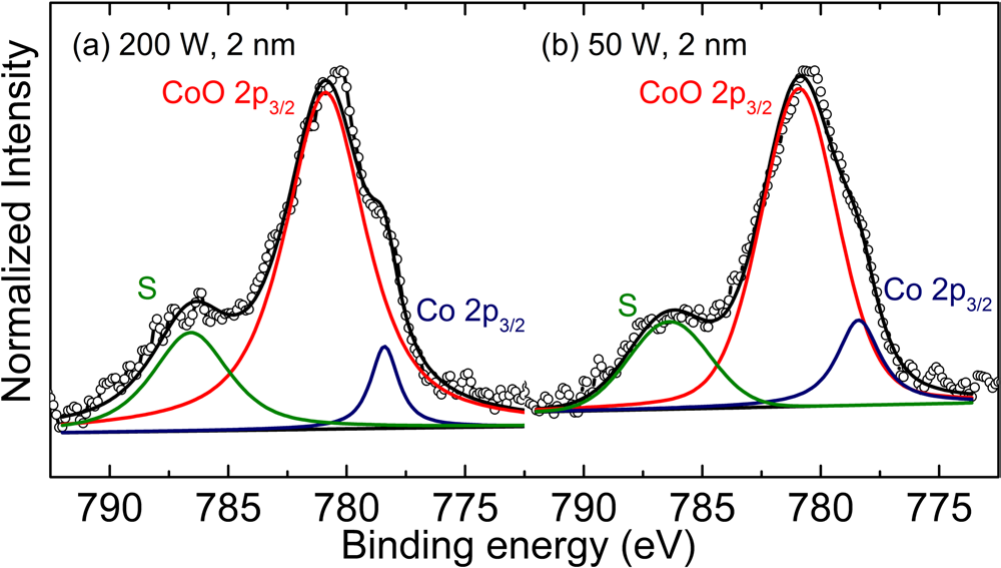
Co 2*p*_3/2_ XPS spectra for the Pt/Co (0.6 nm)/MgO samples at (**a**) (200, 2) and (**b**) (50, 2). The symbol “S” is a satellite peak, which is attributed to the charge transfer between Co 3*d* and O 2*p*.

When the results are compared among the as-deposited samples fabricated at the same conditions, the magnetizations are unusually lower for the Pt/Co (0.6 nm)/MgO samples than for the Pt/Co (1 nm)/MgO ones, indicating that the former samples are contaminated by interpenetrated oxygen atoms to a greater extent than the latter. To confirm this, X-ray photoelectron spectroscopy (XPS) experiments were performed for the as-deposited Pt/Co (0.6 nm)/MgO samples, and the results are shown in Fig. 3. The two peaks, located at 778.4 and 780.9 eV are due to the 2*p*_3/2_ levels of Co from pure Co and CoO, respectively. Other peaks at 786.6 and 786.4 eV denoted by the symbol “S” for the samples at (200, 2) and (50, 2), respectively, are attributed to the charge transfer between Co 3*d* and O 2*p*^9^. The Co 2*p*_3/2_ and CoO 2*p*_3/2_ peaks are fitted with a Gaussian function to estimate their peak areas (*A*), which are then used to calculate the ratio of CoO to metallic Co (*R*_CoO_) using the relation *R*_CoO_ = *A*_CoO_/(*A*_Co_ + *A*_CoO_), where *A*_Co_ and *A*_CoO_ denote the areas of the Co and CoO peaks, respectively^9^. Indeed, the results indicate that the level of oxygen contamination in Co is very high in the as-deposited state, and furthermore, it is higher at (200, 2) than at (50, 2). Specifically, the *R*_CoO_ value is as high as 84% for the sample at (50, 2), and it is further increased to 92% for the sample at (200, 2). These XPS results are in good agreement, at least qualitatively, with the magnetization results.

**Figure 4:**
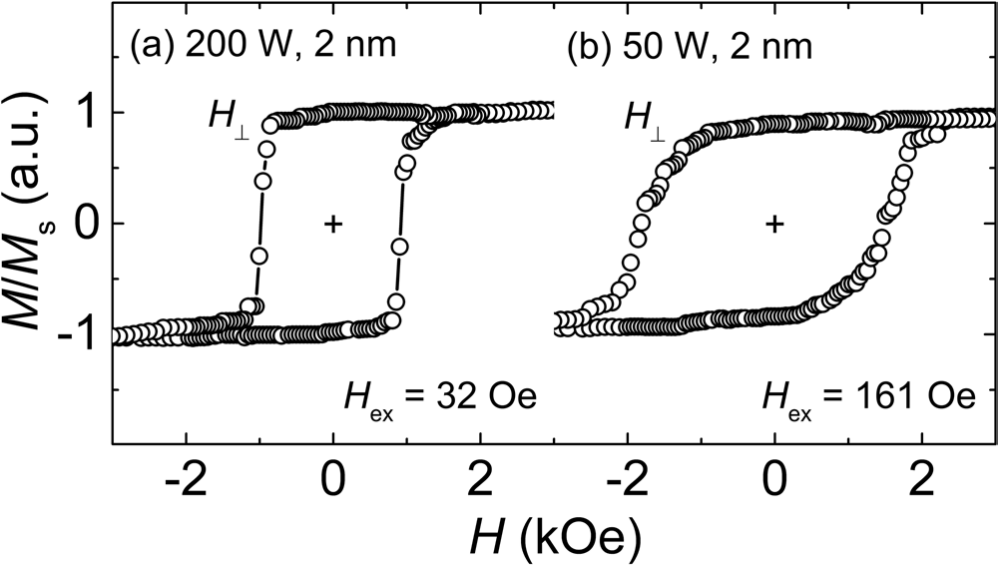
Out-of-plane (*H*_⊥_) *m*-*H* loops measured by SQUID at *T* = 100 K (field-cooled under *H*_⊥_ = + 20 kOe). Each represents the samples of Pt/Co (0.6 nm)/MgO at (**a**) (200, 2) and (**b**) (50, 2).

With such high levels of oxygen contamination in Co, Co oxides such as CoO are expected to be formed in the Co layer, particularly near the Co/MgO interface. In an effort to confirm this possibility, the bias of the hysteresis loops was checked by using a superconducting quantum interference device (SQUID) at a temperature of 100 K, which is lower than the Néel temperature of CoO (293 K)^24^. The as-deposited Pt/Co (0.6 nm)/MgO samples at two different conditions of (200, 2) and (50, 2) were cooled from room temperature to 100 K under an out-of-plane magnetic field of 20 kOe and the hysteresis loops were measured at that temperature. These results are shown in Fig. 4(a) and (b) for the samples at (200, 2) and (50, 2) respectively. It is clear from the results that the loops are displaced from the origin with bias fields of 32 and 161 Oe for the samples at (200, 2) and (50, 2), respectively. It is worth noting that the direction of the bias is opposite to the direction of the applied magnetic field during the cooling of the samples, providing further evidence that the loop shift is due to the exchange bias between Co and CoO. It is rather unexpected to see that the shift is smaller at (200, 2) than at (50, 2); this may be ascribed to a very small amount of metallic Co in the sample at (200, 2) in such a way that it significantly reduces the Co/CoO interface, i.e., the source of the exchange bias.

### PMA properties after annealing

**Figure 5:**
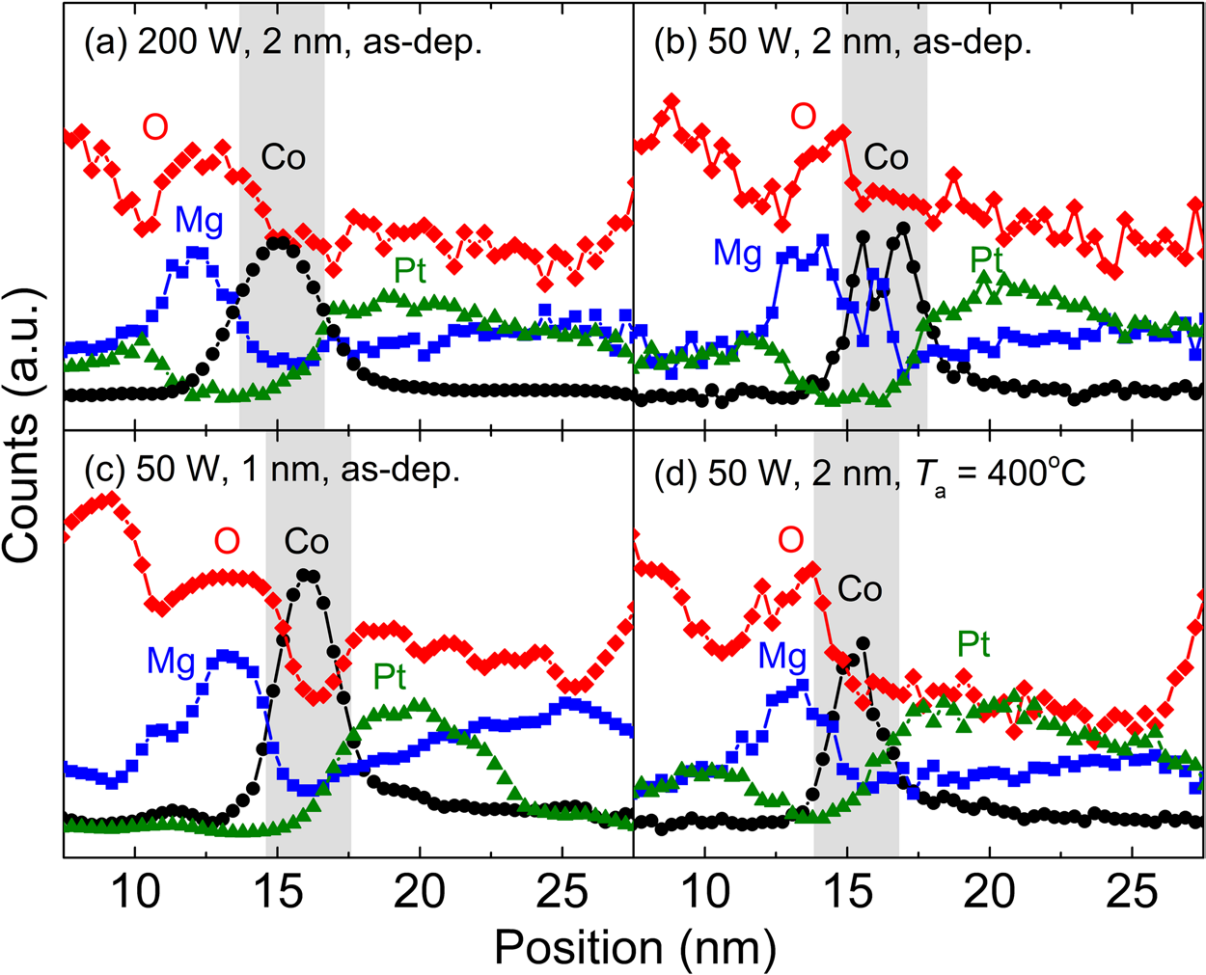
EDS line profiles for the as-deposited Pt/Co (2 nm)/MgO samples fabricated at (**a**) (200, 2), (**b**) (50, 2), and (**c**) (50, 1), and (**d**) for the sample with the same structure fabricated at (50, 2) and then annealed at 400 °C. The Co layer is denoted by the shade.

The PMA properties of all the samples investigated in this study are improved by annealing. This improvement is most probably due to the diffusion of interpenetrated oxygen atoms in the Co layer toward the Co/MgO interface during annealing to form a sharp interface. This is expected owing to the fact that the solubility of oxygen in Co is very limited^25^, and furthermore, in the temperature range of current interest, the Gibbs free energy of formation for MgO is significantly lower than that for Co oxides such as CoO and Co_3_O_4_ (i.e., the equilibrium vapor pressure of O_2_ is significantly lower for MgO than that for Co oxides)^26^. Indeed, this expectation is confirmed by the HRTEM image for the annealed sample shown in Fig. 2(d), where the Co/MgO interface becomes significantly sharper than those for the as-deposited ones (Fig. 2(a–c)). This change in the morphology of the Co/MgO interface on annealing is also supported by the energy dispersive X-ray spectroscopy (EDS) line profiles as shown in Fig. 5(a–d) for the as-deposited Pt/Co (2 nm)/MgO samples fabricated at (200, 2), (50, 2), and (50, 1), and for the sample with the same structure fabricated at (50, 2) and then annealed at 400 °C, respectively. The profile was scanned from the capping layer of Ta (actually Ta/TaO_*x*_ due to the natural oxidation) toward the Si substrate. A comparison of the results shown in Fig. 5(b) and (d) clearly shows the improvement in the quality of the Co/MgO interface on annealing. One prominent feature drawn from the profiles is the reduction of the oxygen content in the Co layer after annealing, with no substantial change in the profiles for the other elements, such as Co, Mg, and Pt. Among the as-deposited samples fabricated at (200, 2), (50, 2), and (50, 1) (Fig. 5(a–c)), the oxygen level is significantly lower for the sample at (50, 1) than that for the samples at (200, 2) and (50, 2), further indicating that the degree of oxygen contamination is very sensitive to *P*_MgO_ and *t*_MgO_.

It is important to note that the PMA properties indicated by the *H*_Keff_, for example, are better for the annealed Pt/Co (1 nm)/MgO stacks than for the Pt/Co (1 nm)/Cu stack. This indicates that after annealing both the Co/MgO and the Pt/Co interfaces start to contribute to the PMA. Since both interfaces now contribute to the PMA, the PMA of annealed samples is indeed very strong; for all the samples shown in Fig. 1, no saturation occurs along the in-plane magnetization curve with a maximum *H* of 19 kOe. The results of SQUID measurements with a maximum *H* of 70 kOe, which are shown in the insets of Fig. 1(a) and (b), show that the saturation field exceeds 40 kOe. It is worth noting that the in-plane magnetization curve is relatively non-linear, indicative of the existence of the second-order PMA.

### Quantitative analysis on degree of oxygen interpenetration

**Figure 6:**
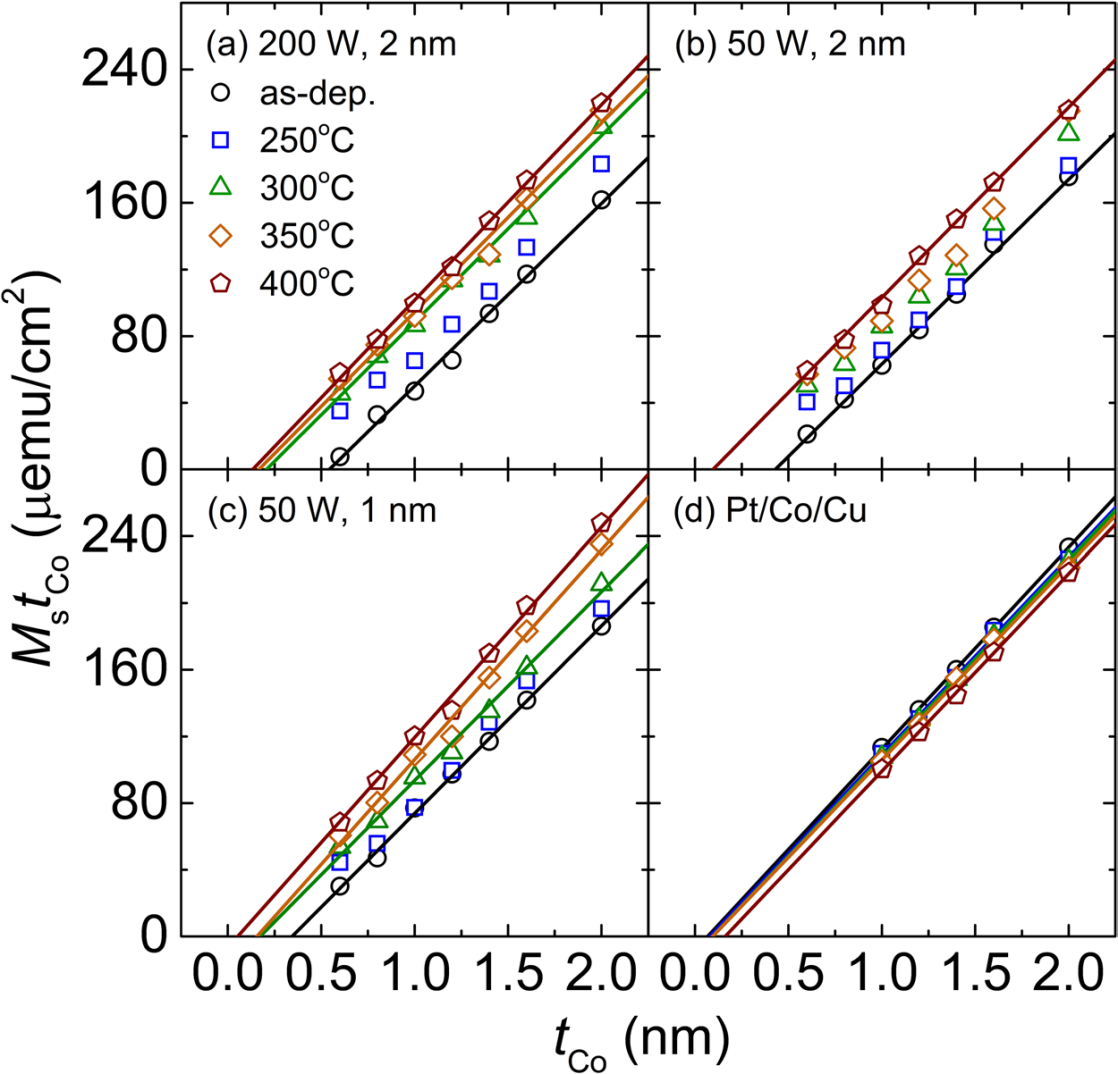
*M*_s_*t*_Co_ vs. *t*_Co_ curves with varying *T*_a_ for the Pt/Co/MgO samples at (**a**) (200, 2), (**b**) (50, 2), (**c**) (50, 1), and (**d**) for the Pt/Co/Cu samples. The lines are the linear fits.

To understand the degree of oxygen contamination in a more quantitative way, the magnetic dead layer (MDL) plots are constructed, and the results are shown in Fig. 6(a–d) for the MgO-containing stacks at (200, 2), (50, 2), (50, 1) and the MgO-free stacks of Pt/Co/Cu, respectively. The results are shown for the samples in the as-deposited state (circles) and also after annealing at 250 °C (squares), 300 °C (triangles), 350 °C (diamonds), and 400 °C (pentagons). Two important parameters of *M*_s_ and *t*_dead_ (where *M*_s_ and *t*_dead_ denote the saturation magnetization and MDL thickness, respectively) can be extracted from the MDL plots—the former from the slope of *M*_s_*t*_Co_ vs. *t*_Co_ and the latter from the extrapolated *t*_Co_ value at which the magnetic moment (or *M*_s_*t*_Co_) is zero. These parameters are summarized in Table 1. When the MDL plots are non-linear, it is impossible to extract the parameters; in this case, the values are missing (cross symbols) in Table 1. The MDL is most probably formed due to the formation of CoO_*x*_ near the Co/MgO interface and the intermixing at the Pt/Co interface. Information on the relative contribution of the two can be estimated by comparing the results of the Pt/Co/MgO stacks with those of the Pt/Co/Cu stacks, where the MDL should be negligible at the Co/Cu interface^21–23^.

**Table 1:** *M*_s_ and *t*_dead_ values extracted from MDL plots

*T*_a_	*M*_s_ (emu/cc)				*t*_dead_ (nm)			
	200 W, 2 nm	50 W, 2 nm	Pt/Co/Cu	50 W, 1 nm	200 W, 2 nm	50 W, 2 nm	Pt/Co/Cu	50 W, 1 nm
as-dep.	1096	1116	1207	1125	0.54	0.43	0.07	0.34
250 °C	×	×	1184	×	×	×	0.08	×
300 °C	1118	×	1184	1132	0.21	×	0.09	0.17
350 °C	1134	×	1170	1257	0.17	×	0.09	0.16
400 °C	1173	1142	1184	1288	0.13	0.10	0.16	0.08

.

It is seen from Fig. 6(a–c) for the as-deposited samples that the MDLs are greater at higher values of *P*_MgO_ and *t*_MgO_ and the same tendency is observed for *M*_s_, though not clearly seen from the plots. The *M*_s_ values are estimated to be 1096, 1116, and 1125 emu/cc for the samples at (200, 2), (50, 2), and (50, 1), respectively. It is worth noting that these *M*_s_ values should be reliable because they were obtained from the MDL plots (from the slope of *M*_s_*t*_Co_ vs. *t*_Co_) over a wide *t*_Co_ range of 0.6 to 2.0 nm. The *t*_dead_ values are 0.54, 0.43, and 0.34 nm for the samples of (200, 2), (50, 2), and (50, 1), respectively. The variation of *t*_dead_ with *P*_MgO_ and *t*_MgO_ can be explained in a manner similar to that of *M*_s_. These changes in *t*_dead_ and *M*_s_ as a function of *P*_MgO_ and *t*_MgO_ are most probably due to similar changes in the oxygen interpenetration. One supporting evidence is that the *M*_s_ value obtained from the as-deposited MgO-free Pt/Co/Cu (1207 emu/cc) is significantly higher than the values from the MgO-containing stacks. The other evidence is that the *t*_dead_ values observed for the Pt/Co/MgO trilayers are significantly higher than the value (0.07 nm) for the MgO-free Pt/Co/Cu.

For the MgO-containing samples at (200, 2), (50, 2) and (50, 1), the *M*_s_ value is increased substantially at the highest annealing temperature (*T*_a_) of 400 °C. On the contrary, an opposite behaviour was observed for the MgO-free stacks of Pt/Co/Cu where the *M*_s_ value was reduced even on annealing. One plausible reason for this is the intermixing during annealing at the interfaces of Pt/Co and Co/Cu. Between the two interfaces, the intermixing is more likely to occur at the bottom Pt/Co interface, because Pt and Co have a strong chemical affinity indicated by a large ordering energy^27^. Considering that the intermixing at the Pt/Co interface will also occur for the Pt/Co/MgO trilayers (thus causing to reduce the *M*_s_ value), the observed increase in *M*_s_ is due to a dominant role of the de-mixing of interpenetrated oxygen atoms from the Co layer.

The *t*_dead_ value decreases progressively with increasing *T*_a_. For the samples at (200, 2), the *t*_dead_ value is 0.54 nm in the as-deposited state and it decreases to 0.13 nm as *T*_a_ increases to 400 °C. A similar behaviour is observed for the sample at (50, 1) where an even lower *t*_dead_ value of 0.08 nm is obtained at the highest *T*_a_ of 400 °C. Unlike the Pt/Co/MgO structures, an opposite tendency is observed for the MgO-free Pt/Co/Cu structure. In this case, the *t*_dead_ value increases minimally up to *T*_a_ of 350 °C, then increases considerably up to 0.16 nm as *T*_a_ reaches 400 °C. These results indicate that for the Pt/Co/Cu stacks, where the MDL mainly occurs at the Pt/Co interface, the intermixing of Pt with Co at the Pt/Co interface is minimal at *T*_a_ up to 350 °C, but starts to occur substantially at 400 °C. At *T*_a_ = 400 °C, the *t*_dead_ value of 0.16 nm observed for the Pt/Co/Cu trilayers is comparable to the value of 0.13 nm for the sample at (200, 2), and is even higher than the value of 0.10 and 0.08 nm for the sample at (50, 2) and (50, 1), respectively. This indicates that at this annealing temperature, the MDL at the Co/MgO interface is minimal with most of the interpenetrated oxygen atoms diffused out from the Co layer to form a clean Co/MgO interface.

It is seen from Fig. 6(a–c) for the Pt/Co/MgO stacks that the MDL plots are non-linear for some of the annealed samples, mainly due to large *M*_s_*t*_Co_ values in the small *t*_Co_ range. One possible reason for the non-linearity is due to the formation of the surface magnetization at the interfaces of Pt/Co^28,29^ and/or Co/MgO^30,31^. With the magnetization formed at the interface, the relative contribution of the surface magnetization to the total magnetization becomes higher at lower *t*_Co_ values, causing the MDL plot to bend. This bending is particularly prominent for the samples at (200, 2), (50, 2), and (50, 1) after annealing at 250 °C. From the observed changes in the *M*_s_ and *t*_dead_ as a function of *T*_a_, the surface magnetization is mainly due to the Pt/Co interface. Considering that the surface magnetization is observed only at a well-defined interface, the quality of the Pt/Co interface is considered best, at least in terms of the surface magnetization, at the annealing temperature of 250 °C^22^. Owing to a strong chemical affinity between Pt and Co, intermixing starts to occur at temperatures higher than this temperature, resulting in the reduction of the surface magnetization.

## Discussion

It is shown that the PMA properties of Pt/Co/MgO trilayers are sensitively dependent on *P*_MgO_ and *t*_MgO_, confirming that they are the key fabrication factors affecting the degree of oxygen interpenetration into the underlying Co layer, and hence, the magnetic properties of the whole structure. When the values of *P*_MgO_ and *t*_MgO_ are high, the interpenetration of oxygen atoms into the Co layer is strong, resulting in poor PMA properties. The EDS depth profiles indicate that the level of interpenetrated oxygen atoms is relatively high, so that both the Pt/Co and the Co/MgO interfaces are susceptible to oxygen contamination, thus deteriorating the PMA properties. This feature is confirmed more clearly by examining the MgO-free model stacks of Pt/Co/Cu. Cross-sectional HRTEM images show that in the as-deposited state, the Co/MgO interface is relatively blurred due to the strong interpenetration of oxygen atoms, thus making its PMA contribution negligible. This means that the PMA properties in the as-deposited state are mostly dependent on the degree of oxygen contamination at the Pt/Co interface. In addition to *P*_MgO_ and *t*_MgO_, the degree of oxygen contamination at the Pt/Co interface is also affected by *t*_Co_; specifically, it is stronger when the *t*_Co_ value is lower. The PMA properties of the as-deposited Pt/Co/MgO stacks improve in a gradual manner by post-annealing at temperatures up to 400 °C examined in this study. This is mainly due to the diffusion of the interpenetrated oxygen atoms in the Co layer toward the Co/MgO interface. This results in a significant change in the morphology of the Co/MgO interface from a blurred to a well-defined geometry, thus allowing the PMA properties to improve. However, the PMA contribution from the Pt/Co interface decreases on annealing, owing to the intermixing of Co and Pt.

## Methods

The stacks examined in this study were Si substrate (wet-oxidized)/Ta (5 nm)/Pt (5 nm)/Co (*t*_Co_)/MgO (1 or 2 nm)/Ta (3 nm), where the Pt/Co/MgO trilayers with the *t*_Co_ value varied from 0.6 to 2.0 nm are the key part exhibiting PMA properties. MgO-free model stacks of Pt (5 nm)/Co (*t*_Co_)/Cu (2 nm) with the same under-layers and capping-layer were also prepared. In the model stacks of Pt/Co/Cu, the *t*_Co_ value was varied from 1.0 to 2.0 nm. All the stacks were fabricated using an ultra-high vacuum magnetron sputtering system consisting of two chambers with base pressures of 5 × 10^−8^ and 1 × 10^−8^ Torr. All the metallic layers were deposited by dc sputtering under an Ar pressure of 2 × 10^−3^ Torr, whereas the MgO layer was grown by RF sputtering at a *P*_MgO_ value of 50 or 200 W under an Ar pressure of 1 × 10^−3^ Torr. The deposition rates of Pt and Co were 0.03 and 0.025 nm/s, respectively, and those of MgO were 0.0054 and 0.035 nm/s at *P*_MgO_ values of 50 and 200 W, respectively. The layer thickness was determined from the deposition rate by controlling the deposition time. The deposition rates of constituent layers were obtained using separately prepared and thick (~100 nm) control samples. To ensure the accuracy of the deposition rates, the thicknesses of control samples were measured for 10 times with a surface profiler (ASIQ, KLA Tencor). In some cases, the thickness of the deposited layers was confirmed by HRTEM (TITAN 80–300, FEI). The difference in the thicknesses from the deposition rate and HRTEM is reasonably small; the *t*_Co_ values obtained from the HRTEM images are 2.15, 2.07, and 2.00 nm for the Pt/Co (2 nm)/MgO samples at (200, 2), (50, 2), and (50, 1), respectively and these values are in good agreement with the designed value of 2 nm (within 7.7%). The layer structure of the stack was also examined using EDS by line profile analysis. In addition, the chemical compositions were investigated by XPS (PHI 5000 VersaProbe, Ulvac-PHI). As-deposited samples were annealed at a vacuum of 9 × 10^−6^ Torr for 30 min at four different *T*_a_ values of 250, 300, 350, and 400 °C. The *m*-*H* loops were measured with a vibrating sample magnetometer (VSM, EV9-380V, Microsense) under out-of-plane and in-plane magnetic fields at room temperature. The VSM results were also used to obtain the MDL plots of *M*_s_*t*_Co_ vs. *t*_Co_. For some samples, the *m*-*H* loops in the high magnetic field range up to 70 kOe were characterized using a SQUID (MPMS EverCool, Quantum Design) at room temperature. Moreover, in some cases, the SQUID measurements were also made at a low temperature of 100 K to check the exchange bias due to the Co/CoO interface that results from the oxidation of the Co layer.
